# The complete mitochondrial genome sequence of *Kolla paulula* from Taiwan

**DOI:** 10.1128/mra.01091-25

**Published:** 2026-02-25

**Authors:** Hsien-Tzung Shih, Chiou-Chu Su, Chung-Jan Chang, Fuh-Jyh Jan, Jianchi Chen

**Affiliations:** 1 Applied Zoology Division, Taiwan Agricultural Research Institute Library 520127 , Taichung, Taiwan; 2 Division of Pesticide Application, Taiwan Agricultural Chemicals and Toxic Substances Research Institute 56085, Taichung, Taiwan; 3 Department of Plant Pathology, University of Georgia 92569, Griffin, Georgia, USA; 4 Department of Plant Pathology, National Chung Hsing University 34916, Taichung, Taiwan; 5 United States Department of Agriculture-Agriculture Research Service, Parlier, California, USA; Indiana University, Bloomington, Indiana, USA

**Keywords:** *Kolla paulula*, mitochondrial genome, insect vector, *Xylella fastidiosa*, Taiwan

## Abstract

The circular mitochondrial genome of *Kolla paulula*, an insect vector of *Xylella fastidiosa* causing grape Pierce’s disease (PD) in Taiwan, is 14,575 bp. The genome encodes genes for 13 proteins, 2 ribosomal RNAs (rRNAs), and 22 transfer RNAs (tRNAs).

## ANNOUNCEMENT

*Kolla paulula* (Hemiptera: Cicadellidae) is a sharpshooter leafhopper commonly found in Taiwan. The insect is a polyphagous, light-to-dark green species with white margins of the fore wings and, in general, not considered a crop pest in Taiwan (1). In 2013, a plant disease, Pierce’s disease (PD) of grapevine, caused by *Xylella fastidiosa* was found in Taiwan (1, 2). The sharpshooter was confirmed to be a vector of *X. fastidiosa* (1, 3). *K. paulula* occurs in all vineyards and is distributed over the medium- and low-altitude areas in Taiwan (1). Current literature shows that the insect is distributed in south and east Asia (4).

Insect mitochondrial genome (mitogenome) is now the most studied genomic system in entomology (5). In GenBank, there are currently two *K. paulula* mitogenome sequences, KY680277 (13,579 bp), a partial mitogenome sequence from Zhenjiang Province of China, and MW542170 (14,995 bp), a complete mitogenome sequence from Guizhou Province of China. Journal publications are not yet available for both records. To facilitate the insect research, we report here a mitogenome sequence of *K. paulula* from Taiwan.

A colony of *K. paulula* was collected and maintained on *Commelina diffusa* under laboratory conditions (25°C, 60% RH [relative humidity], 13:11 L:D [light:day]) at the Taiwan Agricultural Research Institute (Longitude: 120.696404; Latitude: 24.029927). DNA was extracted from a single insect using a DNeasy blood and tissue kit (Qiagen, Valencia, CA). Whole-genomic DNA was amplified using an illustra GenomiPhi version 2 DNA amplification kit (GE Healthcare, Waukesha, WI) and sequenced using NovaSeq (2 × 150) format (Illumina, San Diego, CA) after library preparation using the Illumina TruSeq DNA PCR-free kit.

A total of 15 Giga bp data with average Q value of 36 were generated (FastQC ver. 0.12.1) (6). *De novo* assembly was performed with CLC Genomics Workbench (ver. 24.0.1) using default setting (Word-size = 45; Bubble-size = 98). A contig of 14,617 bp was identified through a BLAST+ (ver. 2.15.0) search using MW542170 as query against the whole *de novo* assembly. Overlaps at the 5′ and 3′ ends were detected by self-BLAST of the contig and edited, resulting in a circular sequence of 14,575 bp (900 × overall coverage, GC% of 33). Sequence annotation was performed using GeSeq ver. 2.03 (7) and Proksee ver. 0.24.1 (8). Protein coding sequences were manually edited and confirmed the start and stop codons based on invertebrate mitochondrial code. The final mitogenome, KPTC1 (*K. paulula* Taichung 1), consists of genes of 13 proteins conserved to typical insect mitogenome, 22 transfer RNAs, and 2 ribosomal RNAs (Table 1).

**TABLE 1:** Selected metrics of mitochondrial genome of *Kolla paulula* KPTC1

Gene (anti-codon)	Type	Location	Size (bp)	Strand	Start codon	Stop codon
trnI(gat)	tRNA	193..258	66	+	–^*a*^	–
trnQ(ttg)	tRNA	256..323	68	−	–	–
trnM(cat)	tRNA	324..393	70	+	–	–
nad2	CDS	394..1362	969	+	ata	taa
trnW(tca)	tRNA	1360..1428	69	+	–	–
trnC(gca)	tRNA	1419..1479	61	−	–	–
trnY(gta)	tRNA	1481..1545	65	−	–	–
cox1	CDS	1550..3085	1536	+	atg	tag
trnL2(taa)	tRNA	3086..3149	64	+	–	–
cox2	CDS	3150..3826	677	+	att	ta−^*b*^
trnK(ctt)	tRNA	3829..3898	70	+	–	–
trnD(gtc)	tRNA	3898..3961	64	+	–	–
atp8	CDS	3970..4113	144	+	ata	taa
atp6	CDS	4110..4757	648	+	ata	taa
cox3	CDS	4758..5537	780	+	atg	taa
trnG(tcc)	tRNA	5537..5598	62	+	–	–
nad3	CDS	5599..5952	354	+	atc	tag
trnA(tgc)	tRNA	5951..6012	62	+	–	–
trnR(tcg)	tRNA	6011..6074	64	+	–	–
trnN(gtt)	tRNA	6072..6140	69	+	–	–
trnS1(gct)	tRNA	6142..6205	64	+	–	–
trnE(ttc)	tRNA	6207..6270	64	+	–	–
trnF(gaa)	tRNA	6270..6332	63	−	–	–
nad5	CDS	6334..8007	1674	−	ttg	taa
trnH(gtg)	tRNA	8008..8069	62	−	–	–
nad4	CDS	8070..9360	1291	−	atg	t−−^*b*^
nad4l	CDS	9384..9665	282	−	atg	taa
trnT(tgt)	tRNA	9668..9731	64	+	–	–
trnP(tgg)	tRNA	9732..9796	65	−	–	–
nad6	CDS	9799..10287	489	+	att	taa
cob	CDS	10280..11416	1137	+	atg	taa
trnS2(tga)	tRNA	11416..11479	64	+	–	–
nad1	CDS	11479..12411	933	−	att	taa
trnL1(tag)	tRNA	12411..12475	65	−	–	–
rrnL	rRNA	12455..13486	1032	−	–	–
–	Repeat Region	13141..13293	153	+	–	–
trnV(tac)	tRNA	13645..13711	67	−	–	–
rrnS	rRNA	13713..14485	773	−	–	–

^*a*^ −, not applicable.

^*b*^ Incomplete termination/stop codon.

A BLASTn search using the KPTC1 as a query against the National Center for Biotechnology Information (NCBI) core_nr database (update date:2025/09/25) identified MW542170.1 as the top hit (Query Coverage = 98%, Percent Identity = 90.25%). However, KY680277, the other Chinese *K. paulula* mitogenome, was distantly related (Query Coverage = 79%, Percent Identity = 73.80%). Meanwhile, over 40 leafhopper mitogenomes from multiple species showed Query Coverage = 98% but Percent Identity ≤80%, for example, *Nanatka castenea* (NC_070000.1, journal citation not available) with Percent Identity of 80.24%.

## Data Availability

This complete mitogenome sequence of KPTC1 has been deposited in DDBJ/EMBL/GenBank under the accession number PX130412. The Illumina read data for the mitogenome are available under BioProject accession number PRJNA1334032 and SRA number SRS26688334. The version described in this paper is the first version, PX130412.1.
